# Rounded atelectasis after exposure to refractory ceramic fibres (RCF)

**DOI:** 10.1186/s12989-021-00441-y

**Published:** 2021-12-29

**Authors:** Ulrike Brueckner, Anne S. Schulze, Dirk Walter, Marian Kampschulte, Joachim Schneider

**Affiliations:** 1 Institute and Outpatient Clinic for Occupational and Social Medicine, Justus-Liebig-University, Aulweg 129, 35392 Giessen, Germany; 2 Institute of Inorganic and Analytical Chemistry, Justus-Liebig-University, Heinrich-Buff-Ring 17, 35392 Giessen, Germany; 3 Clinic for Diagnostic and Interventional Radiology, University Hospital, Justus-Liebig-University, Klinikstrasse 33, 35392 Giessen, Germany

**Keywords:** Aluminium silicate fibres, RCF, Thermal insulation, Rounded atelectasis, Restrictive lung function pattern, Occupational diseases

## Abstract

**Background:**

Refractory Ceramic fibres (RCF) are man-made mineral fibres used in high performance thermal insulation applications. Analogous to asbestos fibres, RCF are respirable, show a pleural drift and can persist in human lung tissue for more than 20 years after exposure. Pleural changes such as localised or diffuse pleural thickening as well as pleural calcification were reported.

**Result:**

A 45 years old man worked in high performance thermal insulation applications using refractory ceramic fibres (RCF) for almost 20 years. During a occupational medical prophylaxis to ensure early diagnosis of disorders caused by inhalation of aluminium silicate fibres with X-ray including high-resolution computed tomography (HRCT), bilateral pleural thickening was shown and a pleural calcification next to a rounded atelectasis was detected. Asbestos exposure could be excluded. In pulmonary function test a restrictive lung pattern could be revealed. In work samples scanning electron microscopy (SEM) including energy dispersive X-ray analysis (EDX) classified used fibres as aluminium silicate fibres. X-ray powder diffraction (XRD) and transmission electron microscopy (TEM) showed crystalline as well as amorphous fibres.

**Conclusions:**

A comprehensive lung function analysis and in case of restrictive lung disorders additional CT scans are needed in RCF exposed workers in accordance to the guidelines for medical occupational examinations comparable to asbestos exposed workers.

## Background

Refractory Ceramic fibres (RCF) are man-made mineral fibres used in high performance thermal insulation applications and mostly to line furnaces, kilns and other industrial heaters. Also RCF are used as thermal barrier in the automotive, marine, petrochemical, steel, aluminium, ceramic, glass and construction industries. RCF are produced by melting (at ~ 1925 °C) a mixture of aluminium oxide (Al_2_O_3_) and silicon oxide (SiO_2_) in approximately proportion or in combination with minor amounts of other inorganic oxides [1]. Therefore the RCF were named also as aluminium silicate fibres. RCF were produced from melting and blowing or spinning process. As manufactured, RCF are in the form of bulk fibres [1]. The diameter of the fibres depends on process parameters. Respirable fibres with the greatest toxicological potential are WHO fibres with a length > 5 µm, a diameter ≤ 3 µm and the length to diameter ratio of at least 3:1. These fibres impede clearance by alveolar macrophages [1]. Lockey et al*.* [2] showed that RCF can persist in human lung tissue for more than 20 years after exposure.

Analogous to asbestos fibres, RCF show a pleural drift. After alveolar deposition the dust particles can trigger a chronic inflammatory reaction in the lung interstitium or be transported in the lymphatic and blood systems. Asbestos fibres can alter the pleura through pleural drift**.** All fibro genic substances have the potential to cause irreversible damage to the lung parenchyma. Pleural changes such as localised or diffuse pleural thickening as well as pleural calcification were reported by LeMaster [3]. Rounded atelectasis after asbestos exposure was described previously [4]. In our opinion this is the first report of rounded atelectasis in connection with calcified pleural plaques following long-term RCF exposure.

## Methods

### Occupational history

In 2000 a 25 year old Caucasian male worker (never smoker) started to work in a RCF processing plant. For the first three years he was employed at a suction station for processing vacuum mouldings by transferring RCF manually from packages into the suction station. He also operated a dry kiln for these vacuum mouldings. After drying the vacuum moulds they had to be cut or sawed to length manually, polished, and holes had to be drilled to attach heating units. The heating units were glued and clenched to the moulds. Finally two half-round moulds were assembled to form one round heating furnace. He was exposed to refractory fibres unprotected during the whole work duration at the suction station, the dry kiln and the final composition of the mouldings (cartridge production). Dust concentrations of RCF were measured at different instants at all workplaces. Since 2004 he was assigned to the cartridge production line also working at the works bench with a lower exposure to RCF. Protective work clothing like protective masks (e.g. FFP2 masks) has not been used during work.

Personalised measurements were taken at different production sectors within the plant in 2012, 2015, 2017 and 2018 as shown in Table 1.

**Table 1:** Personalised measurements of RCFs at different production sectors in Fibres per cm^3^

Production sector	2012	2015	2017	2018
Suction station 1		0.037	0.120	
Suction station 2	0.293	0.118	0.30	
Suction station 3			0.270	0.256
Kiln	0.781	0.492	0.650	0.766
Saw position 1		0.35	0.64	0.30
Saw position 3		1.10	1.21	0.99
Work bench			0.34	0.31
Booth M9		0.11		0.15
Cartridge production				0.32
Final assembly				0.14

## Results

### Clinical history and clinical examination

The worker has been a never smoker and was asymptomatic throughout his entire work life. He never expressed complains of chest pain, dyspnoea or cough. No pulmonary disease (e.g. history of pneumonia, pneumothorax, pleurisy or any other lung disease) has been described before. No complaints were reported while being exposed to fumes, gases, dust or being in wet and cold weather. At the moment the worker is not treated with any medication. A standard posterior-anterior chest radiograph was obtained prior to start of work in November 2000 without any pathological findings. It showed a sharply demarcated diaphragm, free costrophrenic angles, no localised or defuse densities and a normal cardia shadow. He performs endurance sport as running and soccer playing on a regular base. Breath sounds were reduced in the lower left side and the percussion note was dull. Lung expansion was decreased on the left side. Crackles could not be detected. At the moment the patient is not treated with any medication.

### Lung function analysis

In 2018 restrictive lung function was revealed during an occupational medical examination. For grading the pulmonary function, VC, FEV1, TLC, RV, DLCO, DLCO/VA, ITGV, and MEF50 were expressed and analysed as a percent of the predicted value in the reference population (pred.) as recommended by the guidelines GLI 2012 [5–11]. In our outpatient clinic lung function analysis confirmed a reduced vital capacity (VC) of 3.35 L with a lower limit of normal (LLN) of 4.01 L according to GLI 2012. Forced expiratory volume in 1 s (FEV_1_) was reduced with 2.8 L (LLN 3.18 L) whilst FEV1/FVC ratio 82% (LLN 69%) was normal. The diffusing capacity (D_LCO_) of 7.93 mmol/min/KPa (pred. 8.34 mmol/min/KPa) was reduced as well as residual volume divided by total capacity (RV/TLC) 28% (pred.: 41%) and total gas volume (TGV) 2.6 L (pred.: 4.44 L). Predicted oxygen partial pressure at rest was 85.0 mmHg O_2_. Before cardiopulmonary exercise testing, oxygen partial pressure was measured at 81.6 mmHg at rest and 72.9 mmHg at 125 W.

### Radiological findings

**Fig. 1:**
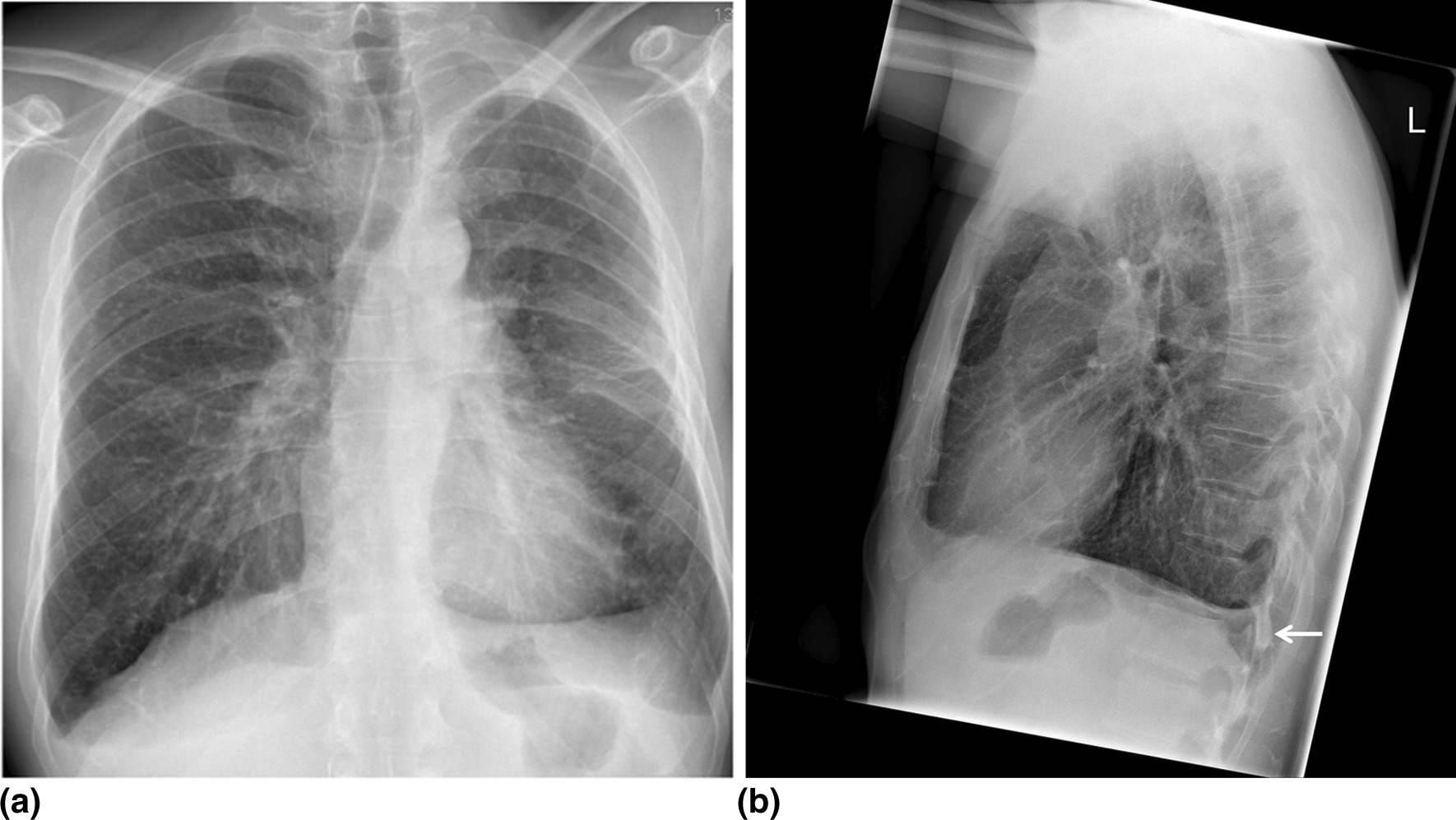
p.a. (**a**) and lateral chest X-ray (**b**) with an adhered costodiaphragmatic sinus (white arrow). Pleural thickening with fibrosis strands is seen on the left middle field

The chest X-rays (p.a. and lateral) showed localised pleural thickening with adhered costodiaphragmatic sinus on the left side and consecutively reduced volume of the left hemi thorax (Fig. 1).

**Fig. 2:**
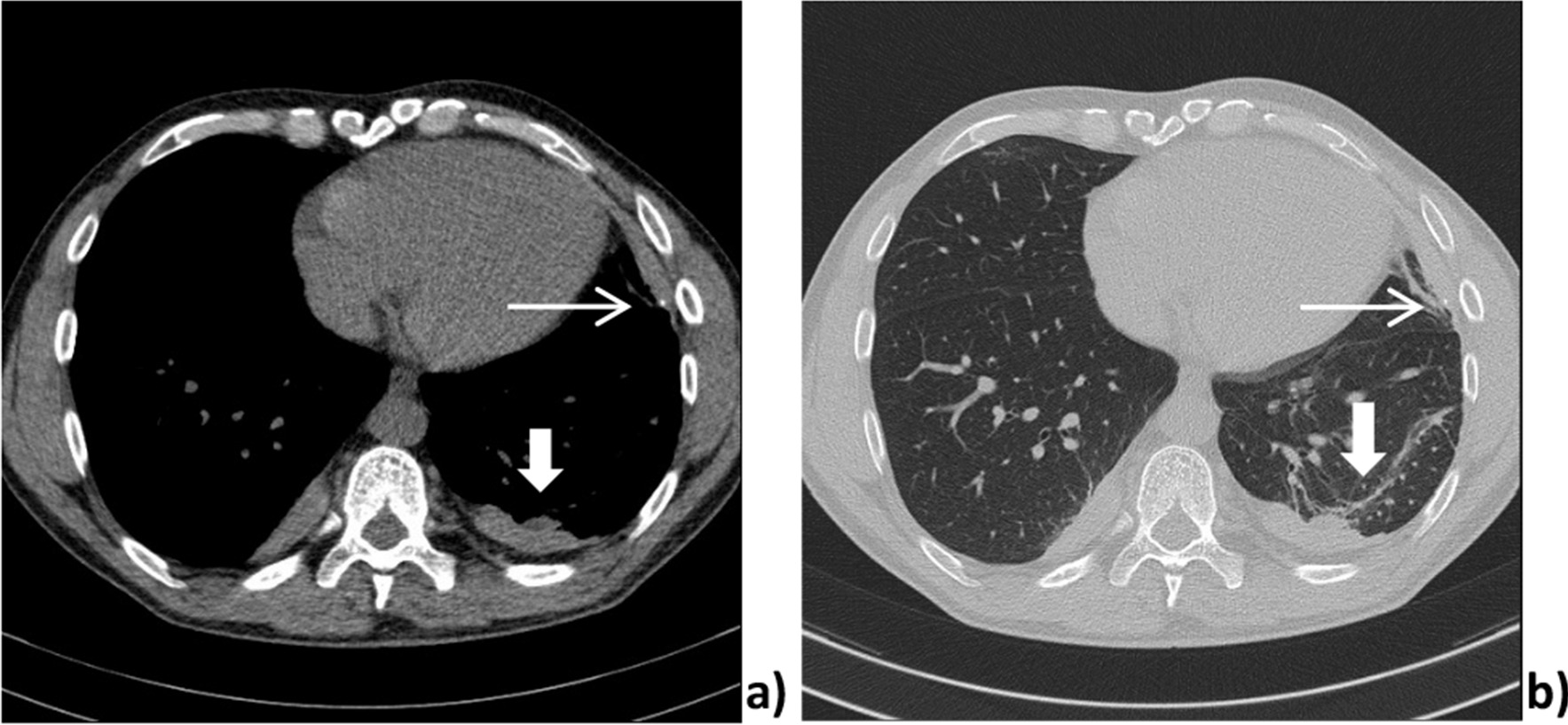
Axial cross sections of the lung-mediastinal window **a**, lung window **b** with diffuse pleural thickening and rounded atelectasis with a “comet tail” sign (thick white arrow) and a reduction in the volume of the left lower lobe. Calcified pleural plaques (slim white arrow)

**Fig. 3:**
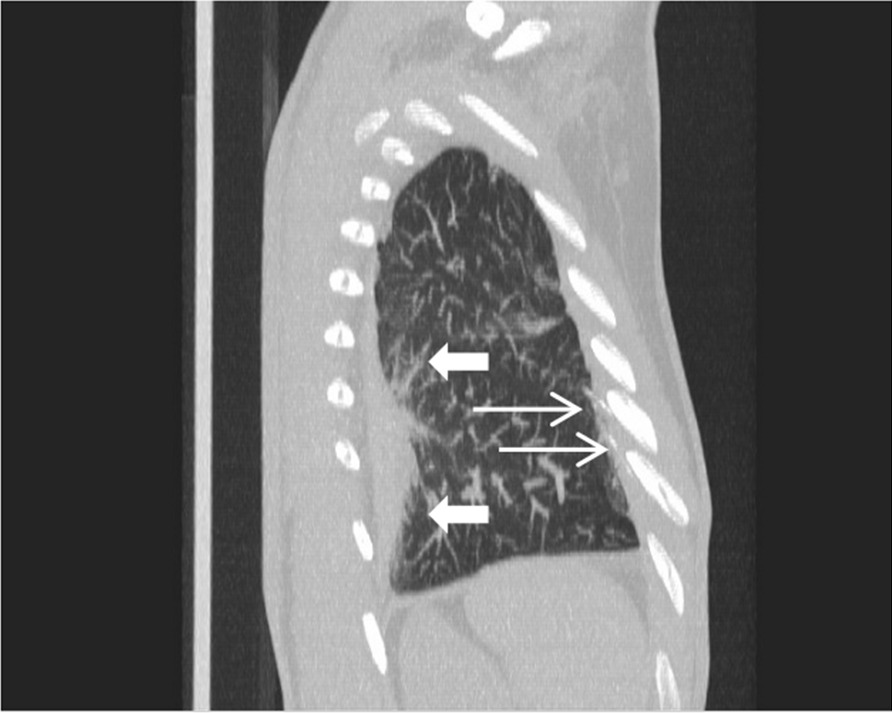
Multiplanar reformation (MPR) of computed lung tomography. Pleural thickening with fibrosis strands on dorsal chest wall (thick white arrows) and pleural calcifications (slim white arrows)

Computer tomography scans presented bilateral pleural thickening especially paravertebral (right side not shown), with embedded pleural calcification only on the left side (Fig. 3). Besides this a beginning rounded atelectasis with a “comet tail” sign is visible adherent to the pleura in the left (Figs. 2, 3). The volume of the left lower lobe (Fig. 2) is reduced.

## Analysis of the insulating material

### Techniques used for the material characterisation

Refractory fibres samples (aluminium silicate fibres) called sample 1, 2 and 3 were obtained from the processing plant and were analysed (raw material RCF: 1a, 2a and 3a; processed RCF from vacuum moulds: 1b, 2b and 3b). Scanning electron microscopy (SEM; Hitachi S-2300; Hitachi, Ltd., Tokyo, Japan) was used to identify fibre geometry in addition to the microstructure of the fibres. To determine the elementary composition Energy Dispersive X-ray spectroscopy (EDX) was applied. To increase the conductivity, all samples were sputtered with a fine layer of Au.

X-ray powder diffraction (XRD) is a common technique to determine the crystal structure of materials. It was used to analyse the crystallinity of the RCF. X-ray powder diffraction in reflection mode was performed with an X’Pert Pro from PANalytical (CuKα radiation (λ = 1.5418 Å), 40 kV, 40 mA). The measurements occurred between 10° and 80° with a step size of 0.033°. With this technique, monochromatic X-ray radiation, generated by a cathode ray tube, creates constructive interference with the sample when the conditions fulfil Bragg’s law:

1 $$n\lambda = 2d \cdot {\text{sin}}\theta$$

Here *n* is an integer, *λ* is the wavelength of the monochromatic X-ray radiation (most common: CuKα radiation λ = 1.5418 Å), *d* is the distance between two lattice planes and *θ* (Theta) is the diffraction angle.

The intensity of the diffracted beam is detected in dependence of the angle 2*θ*, measured in degree (deg), between the incident beam and the detector. The resulting diffraction “peaks” (reflections) can be converted into *d*-spacings, which allows the identification of the material since these *d*-spacings are unique for each compound. While crystalline substances produce a pattern of sharp reflections with different intensities, amorphous compounds only produces a broad background signal. Further information about this technique can be found, for example, in the review article of Bunaciu et al. [12].

**Fig. 4:**
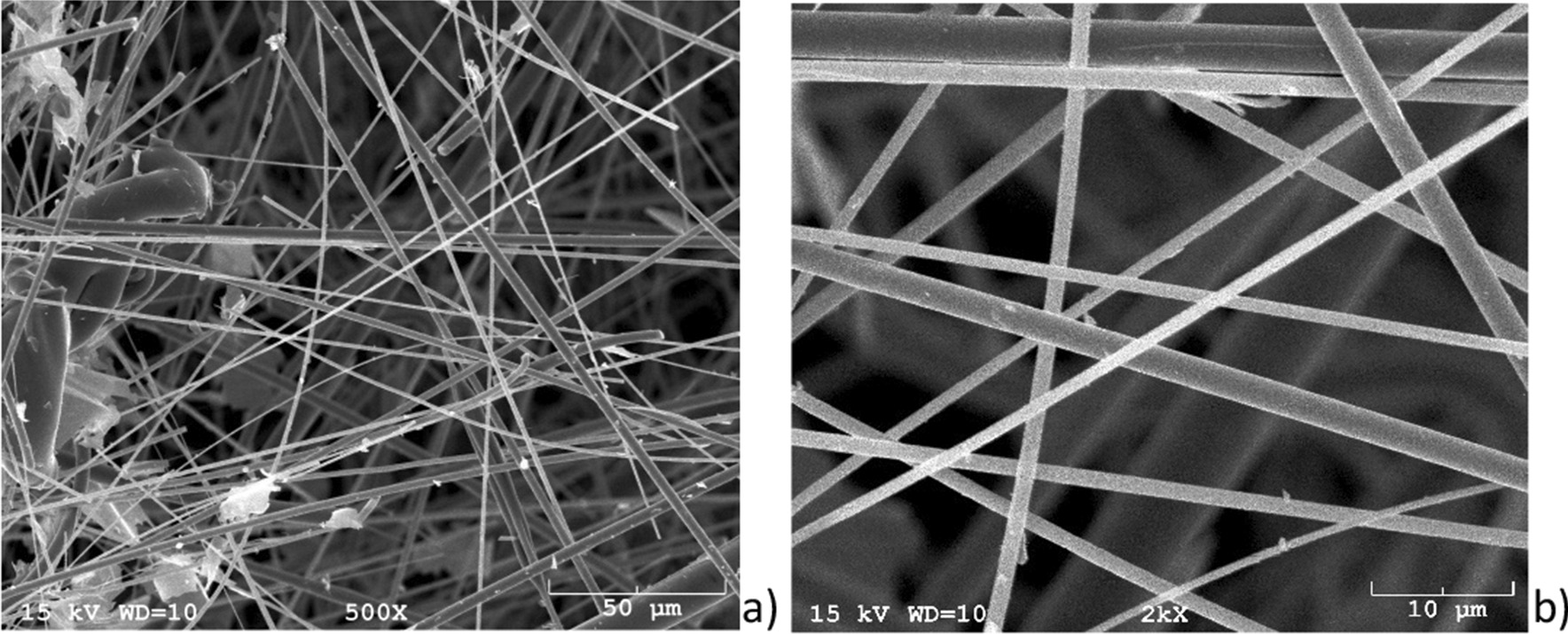
SEM images of the RCF (Magnification 500- (Sample 1a, left (**a**)), 2000-fold (Sample 1a, right (**b**)). Several fibres meet the criteria of WHO fibres

**Fig. 5:**
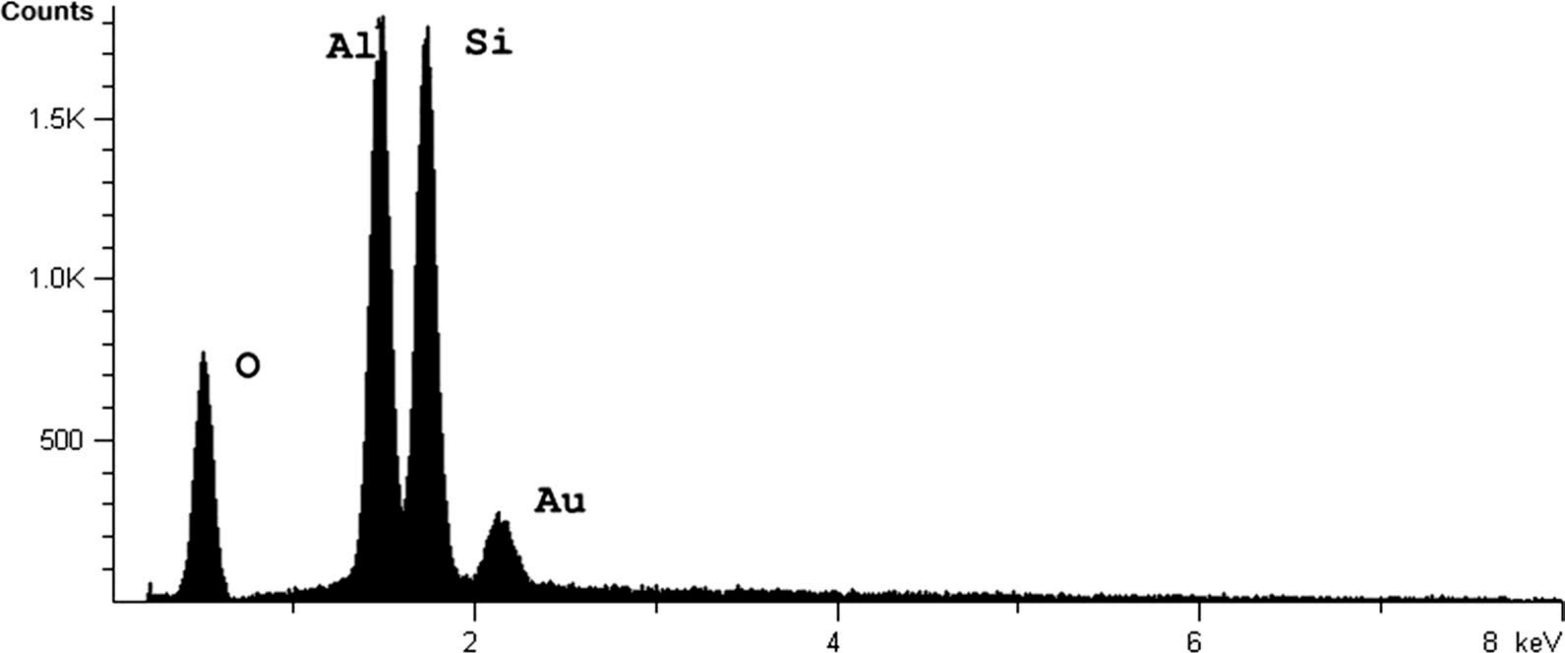
Energy dispersive X-ray spectrum of the RCF Sample 1a as example of aluminium silicate fibres. The EDX spectrum shows oxygen, aluminium and silicon peaks resulting from aluminium oxide (Al_2_O_3_) and silicon dioxide (SiO_2_). The Au peak results from a fine layer of gold from the sample preparation

The crystallinity of the refractory ceramic fibres was additionally investigated with transmission electron microscopy (TEM) and electron diffraction. The TEM images were recorded with a Philips CM30/STEM (300 kV, LaB_6_ cathode) equipped with a GATAN digital camera (Figs. 4, 5).

**Fig. 6:**
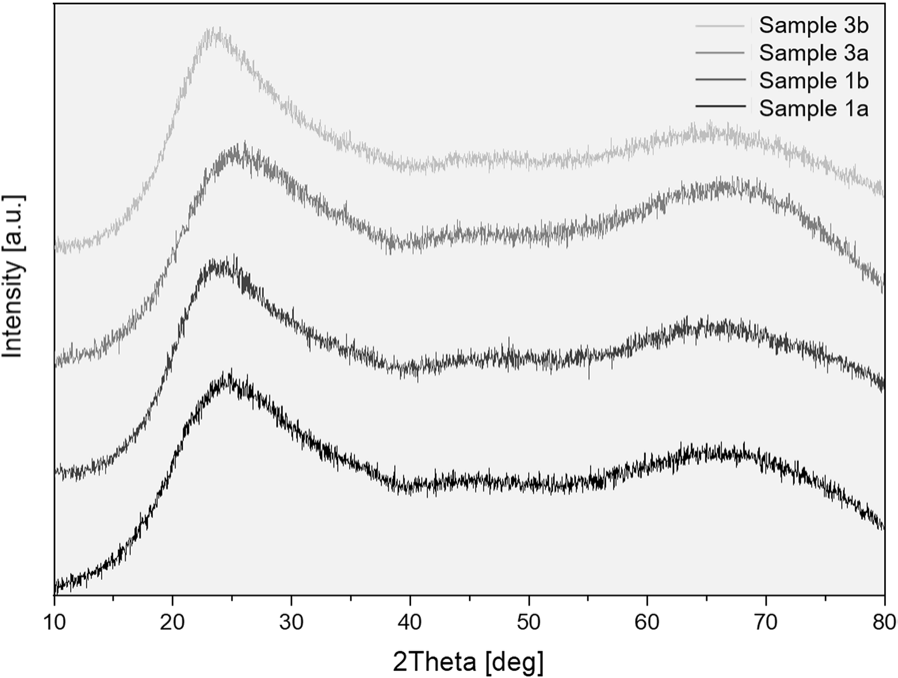
X-ray powder diffraction patterns (normalized; a.u. = arbitrary unit) of the fibres samples 1a, 1b, 3a and 3b. The absence of reflections shows the amorphous character of the samples 1 and 3

In Fig. 6 the results of the X-ray powder diffraction of the samples 1 and sample 3 were presented. The raw material (indicated 1a and 3a) as well as the RCF from processed vacuum moulds (indicated 1b and 3b) showed no reflections, only a broad background signal, which indicates the amorphous character of these samples. The electron diffraction confirms these results; no reflections were visible as well.

**Fig. 7:**
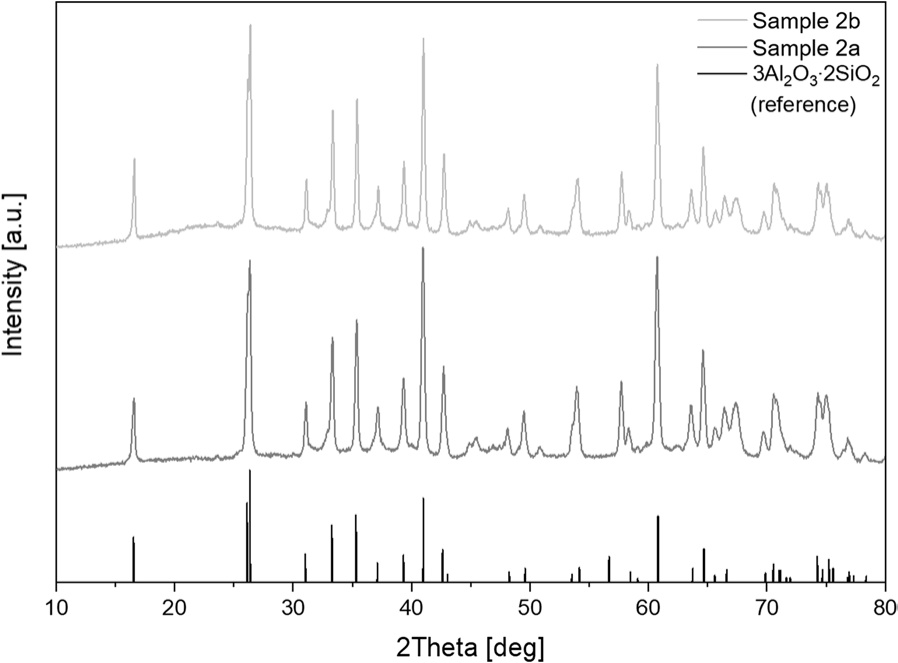
X-ray powder diffraction patterns (normalized; a.u. = arbitrary unit) of the sample 2a and 2b compared to literature data of mullite (3Al_2_O_3_∙2SiO_2_; PDF 00-006-0258)

In contrast, the fibres of the samples 2 are crystalline. The X-ray powder diffraction (Fig. 7) of the sample 2a and 2b show sharp reflections for specific angles, which indicate the crystallinity of these samples. The fibres were identified with a crystallographic data base by the diffraction pattern as mullite (3Al_2_O_3_∙2SiO_2_).

**Fig. 8:**
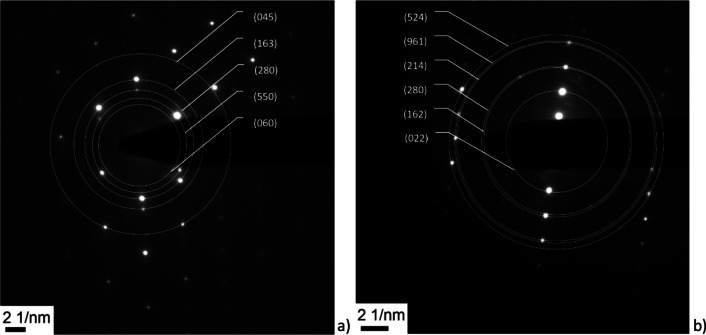
Indexed (Miller indices) electron diffraction images of the sample 2a (left (**a**) and 2b (right (**b**)). The fibres have different crystallographic orientations resulting in a different arrangement of the reflections

The electron diffraction confirms these results. Figure 8 shows the electron diffraction pattern of the samples 2a and 2b. The different arrangement of the reflections originates from different crystallographic orientations of the fibres. The distance between the centre and the reflections can be converted into *d*-spacings, which can be assigned to the Miller indices shown in Fig. 8.

The lattice planes are usually depicted through the Miller indices h, k, l. These three digit numbers describe the orientation of a single lattice plane or a set of parallel planes and result from the points, where a plane cuts the crystallographic axes (a, b, c). The Miller indices are depicted in curvilinear bracket as (hkl). Further information about crystallography can be found, in the review article of Ameh [13].

## Discussion

The safety engineer reported that the worker had been exposed to RCF since 2000. Personalised measurements were taken at different production sectors within the plant in 2012, 2015, 2017 and 2018 and showed elevated concentrations of RCF above 0,3 Fibres/cm^3^ as recommended by European guidelines Directive 2004/37/EC of the European parliament [14] at the saw position 1 and 3, at the dry kiln, at the work bench and the cartridge production. Amorphous and crystalline RCF were detectable during suction processing, drying and in all manual working steps during cartridge production. The fibres did not change physical properties during different processing steps. As shown in Fig. 4 fibres had various lengths with a high proportion of long fibres meeting the WHO criteria as respirable fibres.

During a routine medical examination a restrictive lung function analysis was obvious. In chest X-rays including chest CT scans diffuse pleural thickening, calcifications and a rounded atelectasis could be confirmed. The patient and safety engineer excluded any asbestos exposure at the workplace. The changes were ascribed to RCF exposure at the workplace.

### Animal studies

Animal data indicated there is a risk of pleural changes and malignancies after RCF exposure. Hesterberg and Mast showed in 1995 that exposure to RCF induced lung fibrosis, lung tumours and mesotheliomas in rats and hamsters [15, 16]. RCF exposure over 12 months resulted in macrophage infiltration, bronchiolisation of proximal alveoli, and microgranuloma formation. Additionally Everitt et al. and Gelzleichter et al. found in hamsters increased focal pleural thickenings after a 12-week exposure to RCF [17, 18].

### Symptoms

Data from RCF morbidity in humans indicated that exposed workers suffered from dyspnoe, and showed significant deficiencies in certain measurements in lung function. Also a dose-related increase in pleural plaques was described [3, 19].

Trethowan et al. studied 628 current employees in the manufacturer of ceramic fibres in seven European plants in three countries [20]. In all plants, the most frequent symptoms of the employees were nasal stiffness in 55% of all subjects, 41% complained of eye irritation, 36% complained of skin irritation, 18% of wheeze, 13% dry cough, and 12% fulfilled the criteria of chronic bronchitis. All symptoms were more frequent in current smokers compared with ex or never smokers.

### Lung function changes

The longest ongoing observational study by LeMasters et al. [3], 30-year mortality and respiratory morbidity study of refectory fibres workers, showed localized pleural thickening associated with small decreases in spirometry results. While statistical significance was observed for FVC between cumulative RCF exposure (eg. 15 vs > 60 fibre-months/cc at age 40), there was no consistent pattern demonstrating increasing loss in FVC with higher exposure categories [21, 22]. This was also depended on initial weight and weight gain (*p* < 0.001). Additional FEV1 reduction was associated with cumulative pack-years and current smoking status significantly. Trethowan et al. studied employees in the manufacturer of ceramic fibres [20]. After adjustment for age, sex, height, smoking and past occupational exposure, there was no significant influence in FEV_1_ resp. peakflow in non-smokers. However, there was a significant decrease in FEV_1_ and peakflow in current smokers and ex-smokers. In contrast, Clausen et al. found significantly lower values of FEV_1_ in 340 insulation workers compared with 166 bus drivers [23]. The observed difference was independent of smoking habits and self-assessed former asbestos exposure. In summary RCF exposures failed to be associated with reduced lung function tests so far.

### Pleural changes

Lockey et al. described a dose response between pleural changes and cumulative fibre exposure in RCF workers [2]. In RCF production pleural changes increased after a latency > 20 years with an OR of 10.8 [95% CI: 2.4–47.9] [2, 24] even without any asbestos exposure. In contrast interstitial changes have not been associated with RCF exposure [21, 22, 25]. The occurrence of pleural changes on chest radiographs suggests that RCF have sufficient biopersistence to directly or indirectly induce pleural inflammatory response resulting in pleural thickening [26–28]. LeMasters et al. [3] demonstrated that RCF workers without asbestos exposure had in 6.1% pleural changes, mostly bilateral (67.4%) and localized pleural thickening (LPT) (86.5%) after a latency of 20–30 years. 2.2% showed diffuse pleural thickening (DPT) and 11.2% had both LPT and DPT. Latency categories of RCF exposure were significantly associated with pleural changes: for those in the > 20–30 years latency, the odds ratio (OR) was significant elevated OR = 7.3, [95% CI: 2.0–26.2] and in the > 30 years latency period OR = 7.8 [95% CI: 2.2–27.7].

The new findings of our report are besides findings of pleural thickening and calcified pleural plaques also, the formation of a rounded atelectasis in a RCF worker accompanied with a restrictive lung function. Rounded atelectasis is more common in men (80%) than in women. The most common cause of rounded atelectasis (RA) is occupational exposure to asbestos [29, 30]. The direct mechanism for the development of rounded atelectasis has not been fully explained. According to one of the theories [29], pleural fluid causes local atelectasis due to the pressure on the adjacent lung. If the rate of fluid pleural accumulation exceeds the absorptive capacity of adjacent alveoli, visceral pleura damage occurs with formation of a fissure and translocation of the lung towards that fissure. As a result of this process, the lung folds in a concentric shape maintained by developing adhesions. When the effusion is resorbed, the lung fills in the space around rounded atelectasis. According to another theory, the lesions are initiated by local pleuritis due to agents, such as asbestos fibres. Local accumulation of pleural fluid or fibre dusts in the course of asbestosis, leads to shrinkage and thickening of pleura. The adjacent lung also shrinks and rounded atelectasis develops [31]. Pathophysiological it may be obvious, that not only asbestos fibres but also RCF causes rounded atelectasis.

## Conclusion

A rounded atelectasis was found in chest CT scans in a middle aged worker in a RCF processing plant during a medical check-up. This is accompanied with a restrictive ventilation disorder and reduced diffusing capacity. Meanwhile the restrictive lung disease is accepted as a recognised occupational disease by the accident insurance institution. A comprehensive lung function analysis and in case of restrictive lung disorders additional CT scans are needed in RCF exposed workers in accordance to the guidelines for medical occupational examinations comparable to asbestos exposed workers.

## Data Availability

All data generated or analysed during this study are included in this published article.
